# Children With Special Health Care Needs: An Analysis of National Survey of Children’s Health Database

**DOI:** 10.7759/cureus.59005

**Published:** 2024-04-25

**Authors:** Oroma A Chukuigwe, Emmanuel O Ilori, Ogochukwu Agazie, Umi O Umar, Okelue E Okobi, Tolulope A Fatuki, Raphael S Figueroa, Adaobi E Atueyi, Julio Gonzalez, Miguel Diaz-Miret

**Affiliations:** 1 General Medicine, Sanitas Medical Center, Fulshear, USA; 2 Psychiatry and Behavioral Sciences, Garnet Health Medical Center, Middletown, USA; 3 General Medicine, College of Medicine University of Lagos, Idi Araba, NGA; 4 Family Medicine, Medical Institute of Tambov State University Named After G.R. Derzhavin, Tambov, RUS; 5 Family Medicine, Larkin Community Hospital Palm Springs Campus, Miami, USA; 6 Medicine, Spartan University of Health Sciences, Vieux Fort, LCA

**Keywords:** special healthcare needs, healthcare disparities, demographic patterns, national survey, children's health

## Abstract

Background

Children with Special Health Care Needs (CSHCN) represent a diverse pediatric population requiring healthcare services beyond typical childhood needs. This study analyzes data from the 2016-2020 National Survey of Children's Health Database to elucidate demographic patterns, prevalence rates, and nuanced factors influencing the health and well-being of CSHCN.

Methods

This retrospective observational study focuses on children aged 0-17 who are identified as CSHCN based on Maternal and Child Health Bureau criteria. A comprehensive analysis of the National Survey of Children's Health (NSCH) database examines key variables, including health outcomes, healthcare utilization, parental-reported health status, and socio-demographic factors. Stratified random sampling ensures national representation.

Results

The study encompassed 40,335 patients, revealing that 14.6% (CI: 14.0-15.3, n=6,445) of CSHCN received care in a well-functioning system. Across age groups, 19.1% (CI: 14.0-15.3, n=6,445) of CSHCN aged 0-5 received ongoing treatment, contrasting with 5.7% (CI: 5.2-6.2, n=1,599) in the 12-17 years group. Males exhibited a prevalence of 15% (CI: 14.1-15.9, n=3,674), and females had 14.2% (CI: 13.2-15.2, n=2,771). Racial disparities were noted; non-Hispanic Native Hawaiian/Other Pacific Islander children had a 3% (CI: 1.1-8.1, n=6) prevalence.

Across Federal Poverty Level categories, prevalence ranged from 12.5% (CI: 11.5-13.6, n=1,753) to 17.7% (CI: 16.6-18.9, n=2,856). Notably, 18.5% (CI: 17.4-19.7, n=3,515) of children without adverse experiences were CSHCN. Among CSHCN in two-parent currently married households, 15.9% (CI: 15.0-16.8, n=4,330) received treatment, while those in unmarried households had a prevalence of 12.9% (CI: 10.5-15.7, n=335). CSHCN with parents born in the United States showed a prevalence of 15.4% (CI: 14.7-16.1, n=5,257).

Conclusion

This study provides valuable insights into the prevalence and demographic patterns of CSHCN. Limitations include potential recall bias and the retrospective study design. Despite these constraints, the findings lay a foundation for future research and targeted interventions, fostering a deeper understanding of the evolving landscape of pediatric healthcare in the United States.

## Introduction

Children with special healthcare needs (CSHCN) represent a diverse group requiring healthcare services beyond typical pediatric needs, stemming from chronic physical, developmental, behavioral, and emotional conditions. This encompasses a spectrum of challenges, emphasizing the necessity for tailored healthcare approaches to ensure equitable access to necessary services [1-4]. In the United States, the prevalence of CSHCN is significant, as it impacts millions of children and their families. In 2017-2018, it was estimated that 13.6 million United States children (18.5% of the pediatric population) were identified as CSHCN, implying that the condition impacts one in every four households (24.8%). The challenges faced by these families extend beyond healthcare, as the condition also affects their financial stability and support systems. Despite the potential alleviation of some financial burdens through public insurance expansions, persistent disparities have persisted, necessitating a closer examination of the evolving landscape of healthcare access and outcomes for CSHCN [5].

CSHCN's pathophysiology involves diverse underlying conditions, disrupting normal physiological processes and impacting organ systems. Influenced by genetic, environmental, and biological factors, the affected children may have altered immune responses, neurological abnormalities, and impaired growth [6]. Conditions like neuromuscular disorders necessitate interventions such as continuous positive airway pressure (CPAP) to address compromised respiratory function, optimize oxygen exchange, alleviate respiratory distress, and promote respiratory health [7-9]. Understanding their pathophysiology is crucial for tailoring effective interventions and comprehensive care strategies to mitigate against the effects of the underlying condition on overall well-being [10].

The National Survey of Children's Health (NSCH) serves as a crucial repository of information, providing valuable insights into the health and well-being of children in the United States. This study focuses on the 2016-2020 period, highlighting health disparities, access to care, and overall well-being. By rigorously analyzing this dataset, the research seeks to provide valuable insights into evolving healthcare dynamics for CSHCN, contributing to informed policy development, and improved healthcare outcomes. This endeavor aims to deepen understanding regarding their needs, assess existing support systems' effectiveness, and identify areas for enhancement [11].

## Materials and methods

Study design and data sources

In this retrospective observational study, an analysis was conducted on children aged between 0 and 17 years identified as CSHCN, based on criteria established by the Maternal and Child Health Bureau. The comprehensive examination utilized data from the NSCH database spanning the years 2016 to 2020, focusing on CSHCN. The methodology employed involved the collection and analysis of demographic information, access to health services, substantive health and well-being indicators, and special healthcare needs data for CSHCN and their families.

Data sources

The primary data source for this research was the NSCH, a nationally representative survey conducted by the United States Census Bureau and supported by the Maternal and Child Health Bureau. In 2016, this survey transitioned from a telephone-based format to self-administered web and paper-based questionnaires, enhancing the depth and breadth of information collected on CSHCN. The survey methods have undergone a few alterations since 2016, particularly in the items comprising the measures. These changes include adjustments to wording, modifications in response options, and alterations in skip patterns or question placement. It is important to note that the fundamental concept of the System of Care remains unchanged. These methodological shifts are essential to recognize as they could potentially influence the interpretation of results over the years.

Population and sampling

The study focused on analyzing secondary data collected from individuals aged 0 to 17 years old across all 50 states and the District of Columbia. The data encompassed children across all 50 states and the District of Columbia, aiming to capture the breadth of the United States' demographic diversity. The original data collection, conducted by the NSCH, employed a stratified random sampling method to ensure accurate representation across various demographic factors, including age, gender, race/ethnicity, and socioeconomic status. This approach facilitated the creation of a nationally and state-representative sample for analysis.

Variables

A thorough exploration of socio-demographic variables, such as age, gender, race/ethnicity, household income, parental education, presence of parental nativity, and childhood adverse experiences was undertaken to capture the intricate factors influencing the healthcare needs of CSHCN.

Data analysis

For the data analysis, descriptive statistics was used in characterizing the study population. These included the prevalence rates of CSHCN and their distribution across socio-demographic variables. Frequencies and percentages were also computed using Excel and were used in the description of the counseling trends, even as subgroup analyses based on demographic, socioeconomic, and child health indicators were conducted to identify variations. As part of the weighting process in the original survey, missing data for certain demographic characteristics were imputed.

Ethical considerations

This study did not require ethical approval as it used anonymized public data.

## Results

The study included 40,335 patients during the specified time frame. Overall, the data revealed that 14.6% (CI: 14.0-15.3%, n=6,445) of CSHCN within the sampled population received care in a well-functioning system. The proportion of CSHCN based on the demographic variables is indicated in Table 1.

**Table 1:** Percent of children with special healthcare needs based on demographic variables C.I.- Confidence interval The data have been represented as N, %, Mean±SD.

Demographic characteristics	Variables		Receive care in a well-functioning system	Do not receive care in a well-functioning system
Total	Total surveyed population	N (%)	6445 (14.6)	33,890 (85.4)
		C.I.	14.0 - 15.3	84.7 - 86.0
		Sample Count (N)	6,445	33,890
		Pop. Est.	2,036,607	11,876,551
Data Based on age group	0-5 years old	%	1,198(19.1)	4,613(80.9)
		C.I.	17.2 - 21.1	78.9 - 82.8
		Sample Count (N)	1,198	4,613
	6-11 years old	N (%)	3,648 (23.1)	9,928 (76.9)
		C.I.	21.8 - 24.5	75.5 - 78.2
		Sample Count (N)	3,648	9,928
	12-17 years old	N (%)	1,599 (5.7)	19,349 (94.3)
		C.I.	5.2 - 6.2	93.8 - 94.8
		Sample Count (N)	1,599	19,349
Data based on gender	Male	N (%)	3,674 (15)	19,202 (85)
		C.I.	14.1 - 15.9	84.1 - 85.9
		Sample Count (N)	3,674	19,202
	Female	N (%)	2,771 (14.2)	14,688 (85.8)
		C.I.	13.2 - 15.2	84.8 - 86.8
		Sample Count (N)	2,771	14,688
Data based on race	Hispanic	N (%)	604 (11.8)	3,909 (88.2)
		C.I.	10.1 - 13.7	86.3 - 89.9
		Sample Count (N)	604	3,909
	White, non-Hispanic	N (%)	4,638 (16)	23,601 (84)
		C.I.	15.3 - 16.8	83.2 - 84.7
		Sample Count (N)	4,638	23,601
	Black, non-Hispanic	N (%)	478 (13.2)	2,550 (86.8)
		C.I.	11.5 - 15.1	84.9 - 88.5
		Sample Count (N)	478	2,550
	Asian, non-Hispanic	N (%)	160 (15.1)	1,040 (84.9)
		C.I.	10.3 - 21.6	78.4 - 89.7
		Sample Count (N)	160	1,040
	American Indian/Alaska Native, Non-Hispanic	N (%)	39 (11.1)	254 (88.9)
		C.I.	7.2 - 16.7	83.3 - 92.8
		Sample Count (N)	39	254
	Native Hawaiian/Other Pacific Islander, Non-Hispanic	N (%)	6 (3)	75 (97)
		C.I.	1.1 - 8.1	91.9 - 98.9
		Sample Count	6	75
	Multiple race, non-Hispanic	N (%)	504 (17.5)	2,340 (82.5)
		C.I.	15.1 - 20.3	79.7 - 84.9
		Sample Count (N)	504	2,340

Based on age group

**Figure 1:**
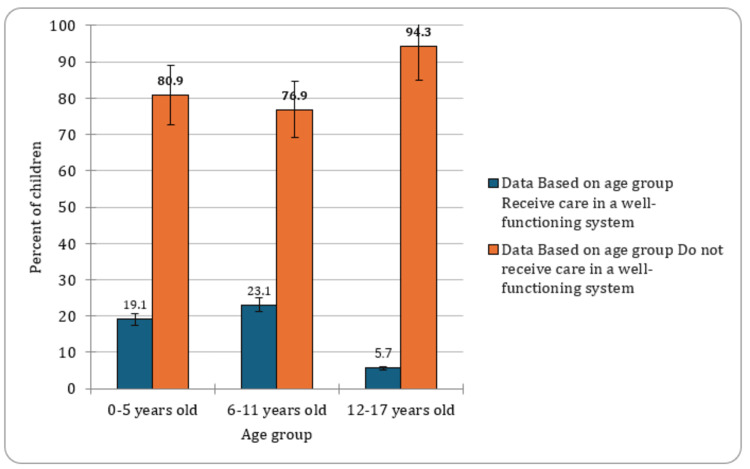
Percent of children with special healthcare needs based on age groups

The analysis of CSHCN data revealed distinct patterns based on age groups. Among CSHCN aged 0-5 years, 19.1% (CI: 14.0-15.3, n=6,445) reported receiving ongoing treatment, while in the 12-17 years age group, the percentage increased to 5.7% (CI: 5.2-6.2, n=1,599). Notably, the highest treatment utilization was observed among CSHCNs aged 6-11 years, with 23.1% (CI:21.8-24.5, n=3,648) receiving necessary care. These findings suggested an age-dependent trend in accessing healthcare services, with older children exhibiting higher treatment rates. Understanding these age-specific nuances was crucial for developing targeted interventions to address the diverse needs of CSHCN across different developmental stages. The graphical presentation of the proportion of children with special healthcare needs based on age groups is indicated in Figure 1.

Based on gender**

**Figure 2:**
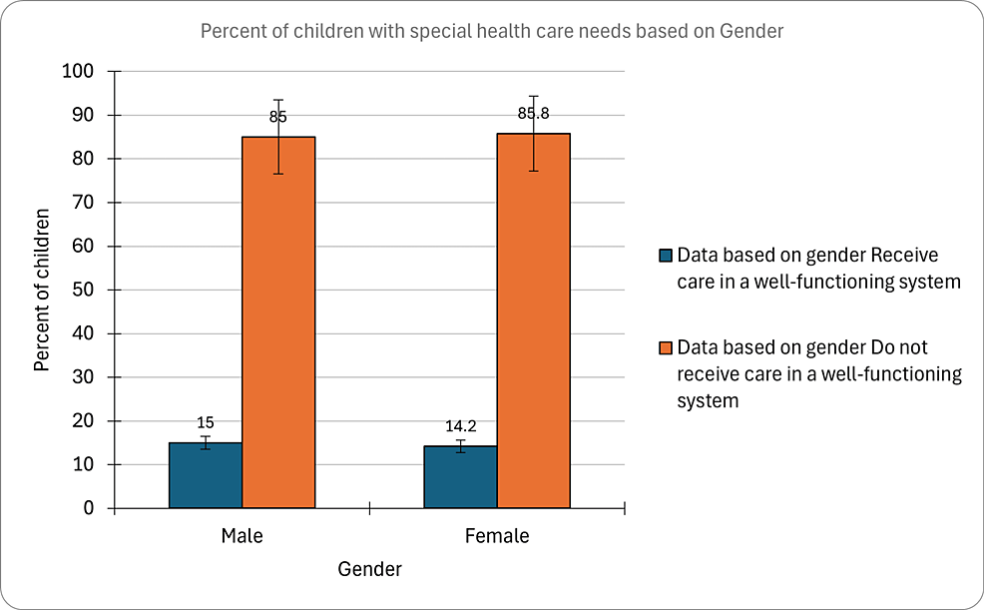
Percent of children with special healthcare needs based on gender

Among the sampled population, 15% (CI: 14.1-15.9, n=3,674) of males were identified as CSHCN, whereas 14.2% (CI: 13.2-15.2, n=2,771) of females fell within this category. This gender-specific breakdown underscored a marginal numerical disparity, suggesting a nuanced aspect of healthcare needs within the studied cohort. Further exploration of these findings might provide valuable insights into potential gender-related factors influencing the prevalence of special healthcare needs among children (Figure 2).

Based on race

**Figure 3:**
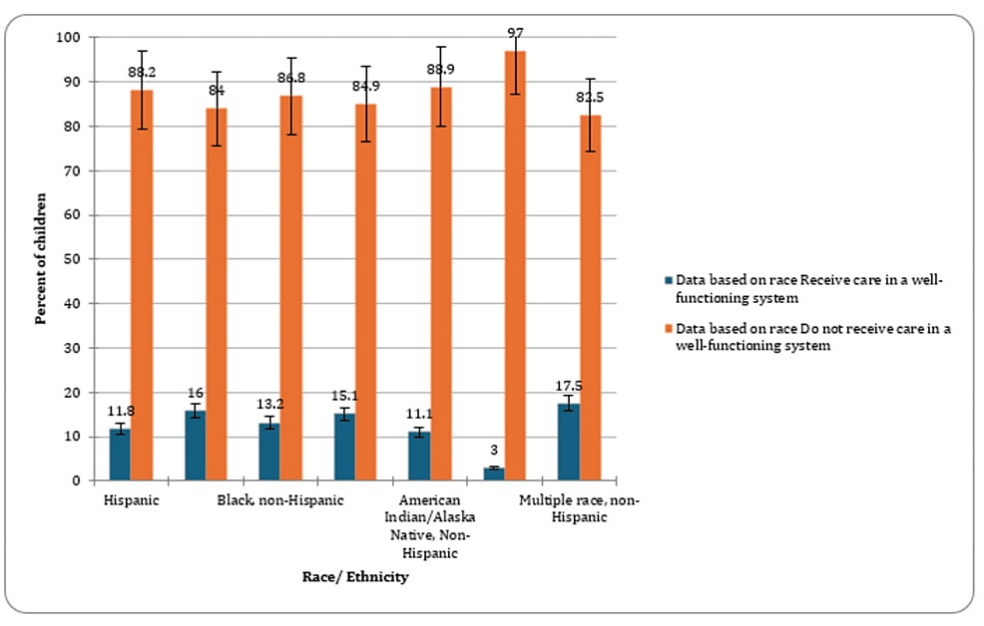
Percent of children with special healthcare needs based on race

The percentage of CSHCN varied among different racial categories. Among non-Hispanic Native Hawaiian/Other Pacific Islander children, 3% (CI: 1.1-8.1, n=6) were identified as having special healthcare needs. In the non-Hispanic American Indian/Alaska Native group, the prevalence was 11.1% (CI: 7.2-16.7, n=39), while among Hispanic children, it was 11.8% (CI: 10.1-13.7, n=604). Non-Hispanic Black children exhibited a higher prevalence of 13.2% (CI: 11.5-15.1, n=478), and non-Hispanic Asian children had a prevalence of 15.1% (CI: 10.3-21.6, n=160). Non-Hispanic White children had a special healthcare needs prevalence of 16% (CI: 15.3-16.8, n=4,638), and those of multiple race, non-Hispanic, had the highest prevalence at 17.5% (CI: 15.1-20.3, n=504). These findings highlighted the racial disparities in the prevalence of special healthcare needs among children. A graphical presentation of the proportion of CSHCN based on race has been provided in Figure 3.

Based on the federal poverty level 

**Figure 4:**
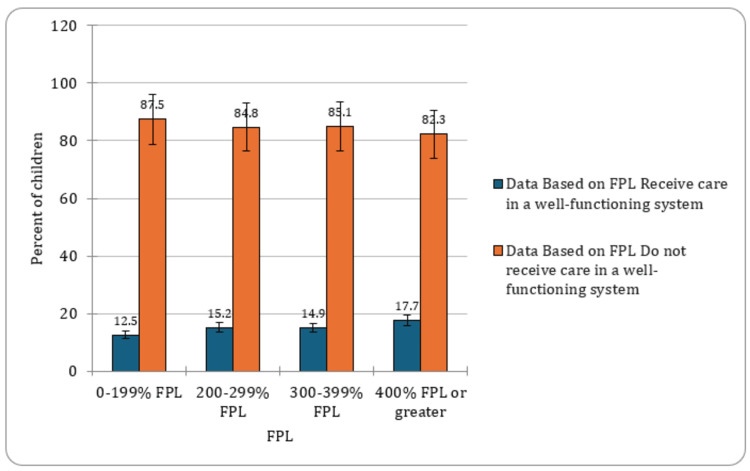
Percent of children with special healthcare needs based on FPL FPL- Federal Poverty Level

The findings revealed varying percentages among different FPL categories. In the 0%-199% FPL range, 12.5% (CI: 11.5-13.6, n= 1,753) of children were identified as CSHCN. The prevalence increased to 15.2% (CI: 13.5-17.0, n=945) for the 200%-299% FPL range, followed by 14.9% (CI: 13.4-16.5, n=891) for the 300%-399% FPL range (Figure 3). Among children in families with an FPL of 400 or more, the prevalence was highest at 17.7% (CI: 16.6-18.9, n=2,856). These results underscored the impact of socioeconomic factors on the prevalence of special healthcare needs among children. A graphical presentation of the percentage of CSHCN based on the federal poverty level has been provided in Figure 4.

Based on childhood adverse experiences

Notably, 18.5% (CI: 17.4-19.7, n=3,515) of children with no reported adverse experiences were identified as CSHCN. In contrast, the prevalence decreased to 13.3% (CI: 12.1-14.6, n=1,320) among those with one reported adverse experience. Furthermore, children who experienced two or more adverse events exhibited a lower prevalence of CSHCN at 11.3% (CI: 10.3-12.4, n=1,540). These findings suggested a potential correlation between the number of childhood adverse experiences and the likelihood of developing special healthcare needs in children. The proportion of CSHCN based on socioeconomic and child health index variables has been presented in Table 2.

**Table 2:** Percent of children with special healthcare needs based on socioeconomic and child health index variables C.I,- Confidence interval, FPL- Federal Poverty Level The data have been represented as N, %, Mean±SD.

Socioeconomic and child health index characteristics	Variables		Receive care in a well-functioning system	Do not receive care in a well-functioning system
Data Based on FPL	0-199% FPL	N (%)	1,753 (12.5)	10,722 (87.5)
		C.I.	11.5 - 13.6	86.4 - 88.5
		Sample Count (N)	1,753	10,722
	200-299% FPL	N (%)	945 (15.2)	5,499 (84.8)
		C.I.	13.5 - 17.0	83.0 - 86.5
		Sample Count (N)	945	5,499
	300-399% FPL	N (%)	891 (14.9)	4,780 (85.1)
		C.I.	13.4 - 16.5	83.5 - 86.6
		Sample Count (N)	891	4,780
	400% FPL or greater	N (%)	2,856 (17.7)	12,889 (82.3)
		C.I.	16.6 - 18.9	81.1 - 83.4
		Sample Count (N)	2,856	12,889
Data based on adverse experiences	No adverse childhood experiences	N (%)	3,515 (18.5)	14789 (81.5)
		C.I.	17.4 - 19.7	80.3 - 82.6
		Sample Count (N)	3,515	14,789
	One adverse childhood experience	N (%)	1,320( 13.3)	8,026 (86.7)
		C.I.	12.1 - 14.6	85.4 - 87.9
		Sample Count (N)	1,320	8,026
	Two or more adverse childhood experiences	N (%)	1,540 (11.3)	10,625 (88.7)
		C.I.	10.3 - 12.4	87.6 - 89.7
		Sample Count (N)	1,540	10,625
Based on parent status	Two parents, currently married	N (%)	4,330 (15.9)	21,542 (84.1)
		C.I.	15.0 - 16.8	83.2 - 85.0
		Sample Count (N)	4,330	21,542
	Two parents, not currently married	N (%)	335 (12.9)	2,148 (87.1)
		C.I.	10.5 - 15.7	84.3 - 89.5
		Sample Count (N)	335	2,148
	Single parent (single mother in 2016)	N (%)	1,138 (12.5)	7,272 (87.5)
		C.I.	11.3 - 13.9	86.1 - 88.7
		Sample Count (N)	1,138	7,272
	Other	N (%)	544 (15.8)	2,309 (84.2)
		C.I.	13.5 - 18.3	81.7 - 86.5
		Sample Count (N)	544	2,309
Based on birth place of parents	Parent(s) born in the US	N (%)	5,257 (15.4)	27,236 (84.6)
		C.I.	14.7 - 16.1	83.9 - 85.3
		Sample Count (N)	5,257	27,236
	Any parent born outside of US	N (%)	609 (12.2)	3,986 (87.8)
		C.I.	10.3 - 14.4	85.6 - 89.7
		Sample Count (N)	609	3,986
	Children born in the US, live with caregiver(s) other than parents	N (%)	524 (14)	2,288 (86)
		C.I.	11.9 - 16.3	83.7 - 88.1
		Sample Count (N)	524	2,288

Based on living with parents

**Figure 5:**
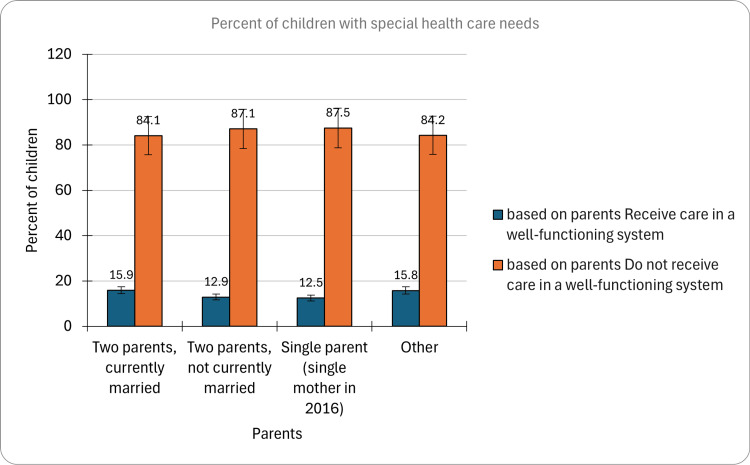
Percent of children with special healthcare needs based on living with parents

Among CSHCN living with two parents currently married, the highest percentage of children receiving treatment was observed, with 15.9% (CI: 15.0-16.8, n=4,330) receiving treatment, while those with two parents not currently married had a treatment prevalence of 12.9% (CI: 10.5-15.7, n=335). Single-parent households, specifically single mothers in 2016, exhibited the lowest percentage of children receiving treatment, with a prevalence of 12.5% (CI: 11.3-13.9, n=1,138). Additionally, children in other living arrangements demonstrated a treatment prevalence of 15.8% (13.5-18.3, n=544). These findings underscored the influence of parental marital status on the access and utilization of healthcare services for children with special healthcare needs. The graphical presentation of the percentage of CSHCN based on living with parents is indicated in Figure 5.

Based on parental nativity

**Figure 6:**
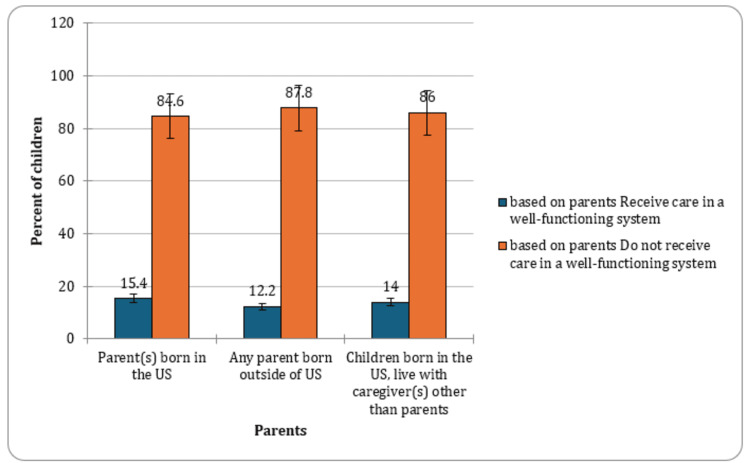
Percent of children with special healthcare needs based on parent status

The examination of the Database revealed variations in the prevalence of CSHCN based on parental nativity. Among children with parents born in the United States, 15.4% (CI: 14.7-16.1, n=5,257) were identified as CSHCN. In contrast, those with at least one parent born outside of the United States had a slightly lower prevalence at 12.2% (CI: 10.3-14.4, n=609). Notably, children born in the United States but residing with caregivers other than their parents exhibited a prevalence of 14% (CI: 11.9-16.3, n=524). These findings underscore the impact of parental nativity on the prevalence of special healthcare needs among children, providing insights for targeted interventions and support strategies. The graphical presentation of the percentage of CSHCN based on parental nativity is indicated in Figure 6.

## Discussion

The analysis of CSHCN data from the NSCH Database unveiled important patterns across various demographic factors, providing valuable insights into the landscape of pediatric healthcare in the United States. The age-dependent trends in accessing healthcare services among CSHCN presented a noteworthy observation. The higher treatment utilization among children aged 6-11 years, as compared to those in the 0-5 and 12-17 years age groups, suggested a critical period for healthcare intervention. Understanding and addressing the evolving needs of CSHCN across different developmental stages were imperative for targeted healthcare strategies. Consistent with earlier research, there was a notable rise in the percentages of youth undergoing healthcare transition. Specifically, the proportion of youths receiving essential healthcare transition preparation saw a significant increase over the study duration, climbing from 14.8% in 2016 to 20.5% in 2019 [12].

The marginal numerical disparity observed in the prevalence of CSHCN between males and females warranted further exploration into gender-related factors influencing healthcare needs within the studied cohort. As per prior research, children and youth with special healthcare needs (CYSHCN) exhibited a higher likelihood of being male compared to their non-CYSHCN counterparts. This insight may contribute to a more nuanced understanding of pediatric health disparities and inform tailored interventions [13].

Racial disparities in the prevalence of special healthcare needs among children underscored the need for culturally sensitive and inclusive healthcare policies. As per previous studies, white CSHCN were found to have a higher likelihood of possessing very good/excellent overall health and oral health compared to Latino CSHCN. Acknowledging these variations can guide the development of equitable interventions to address the diverse healthcare requirements of children from different racial backgrounds [14].

The impact of socioeconomic factors on the prevalence of CSHCN was evident in the varying percentages among different FPL categories. Higher prevalence rates among children in families with lower income levels emphasized the urgency of addressing socioeconomic inequalities to ensure equitable access to healthcare services for all CSHCN. According to previous studies, in the cross-sectional study, the likelihood of underinsurance varied among CSHCN. Both medical complexity and daily functional limitations increased the odds of underinsurance. In households earning 200%-399% of the federal poverty level, underinsurance was linked to complex physical conditions (OR, 2.74; 95% CI, 2.13-3.51) and mental/behavioral conditions (OR, 2.21; 95% CI, 1.87-2.62). In households earning 400% or more, mental/behavioral conditions were associated with underinsurance (OR, 3.31; 95% CI, 2.82-3.88) [15].

The findings of this study suggest a potential inverse relationship between ACE and the prevalence of CSHCN. Specifically, as the number of reported adverse experiences increases, there appears to be a decrease in the prevalence of CSHCN. This trend could indicate a complex interplay between adverse experiences and health outcomes, highlighting the importance of further exploration into the underlying mechanisms and potential protective factors. Addressing adverse experiences early on may prove instrumental in preventing or mitigating the development of special healthcare needs in children. According to a previous study, children and youth with special healthcare needs were more prone to each measured ACE and had a higher likelihood of experiencing cumulative ACEs than non-CSHCN. The probability of CSHCN having a family-centered medical home decreased with rising ACEs, and one or more ACEs heightened the likelihood of unmet mental healthcare needs [16].

The influence of parental marital status on the access and utilization of healthcare services for CSHCN was a critical consideration. Understanding the dynamics of different family structures could inform interventions tailored to the unique needs of children in diverse living arrangements. Moreover, the variations in the prevalence of CSHCN based on parental nativity underscored the complex interplay between cultural and environmental factors. Tailoring interventions to address the distinct needs of children based on their parents' nativity could contribute to more effective healthcare strategies. As per a previous study, CSHCNs with foreign-born parents demonstrated a lower likelihood of having a medical home than those with United States-born parents (adjusted odds ratio = 0.40, 95% CI 0.19-0.85). The adjusted prevalence of having a medical home was 28% for foreign-born CSHCNs and 37% for CSHCNs with a foreign-born parent, contrasting with 49% for CSHCNs with United States-born parents [17].

The study's strength lies in its rigorous design and comprehensive data sources. Utilizing the NSCH data enabled a thorough examination of various indicators. Transitioning to self-administered questionnaires enhanced data collection. Robust sampling ensured a nationally and state-representative sample, enhancing generalizability. Analysis of critical variables like socio-economic demographics offered valuable insights into healthcare needs determinants. Several limitations should be considered when interpreting the findings of this study. Firstly, the data was collected through secondary analysis and primary data also relied on self-reported information provided by parents or caregivers in the original survey, introducing the potential for recall bias. Parents may not have accurately remembered or reported the health conditions and treatment status of their children, leading to inaccuracies in prevalence estimates. Additionally, the study design was cross-sectional, limiting the ability to establish causal relationships between demographic factors and the prevalence of CSHCN. Longitudinal data would have provided a more comprehensive understanding of the dynamic nature of healthcare needs over time. Furthermore, the survey might not have captured the full spectrum of CSHCN, as some cases may have been undiagnosed or not disclosed by caregivers. The NSCH Database focused on the civilian, noninstitutionalized population, potentially excluding CSHCN in institutional settings. The study's temporal scope (2016-2020) may not have captured recent developments or accounted for potential changes in healthcare access and utilization patterns. Finally, while efforts were made to control for various demographic variables, the complexity of factors influencing healthcare needs in children may not have been fully captured, warranting caution in generalizing the findings to all pediatric populations. Despite these limitations, this study contributed valuable insights into the prevalence and demographic patterns of CSHCN, laying the groundwork for future research and targeted interventions.

## Conclusions

In conclusion, the 2016-2020 NSCH Database analysis provides critical insights into CSHCN in the United States. The analysis of CSHCN data uncovered significant patterns across demographic factors. Notably, older children exhibited higher treatment rates, with those aged 6-11 years demonstrating the highest utilization. Gender differences were marginal, with a slightly higher prevalence among males compared to females. Racial and socioeconomic disparities were evident, with Multiple race, non-Hispanic, Asian, and Black children showing higher prevalence rates. Additionally, children from families with higher FPL had increased prevalence. Furthermore, the analysis highlighted a potential inverse relationship between adverse childhood experiences and the prevalence of CSHCN, underscoring the importance of understanding and addressing these complex dynamics in healthcare access. Acknowledging study limitations, longitudinal research is recommended to establish causal relationships and comprehend the dynamic nature of healthcare needs over time. These findings emphasize the need for targeted interventions to address disparities and ensure equitable healthcare for all CSHCN.
